# The effects of different iron shaft weights on golf swing performance

**DOI:** 10.3389/fbioe.2024.1343530

**Published:** 2024-02-06

**Authors:** Chia-Chen Yang, Che-Chia Chang, Te Chao, Hsia-Ling Tai, Yung-Shen Tsai

**Affiliations:** ^1^ Graduate Institute of Sports Training, University of Taipei, Taipei, Taiwan; ^2^ Graduate Institute of Sports Science, University of Taipei, Taipei, Taiwan; ^3^ Department of Physical Education, University of Taipei, Taipei, Taiwan; ^4^ Graduate Institute of Sports Equipment Technology, University of Taipei, Taipei, Taiwan

**Keywords:** handicap, motion analysis, biomechanics, weight transfer, launch monitor

## Abstract

This study examined the effects of three 7-iron shaft weights on golf swing performance among golfers of varying skill levels. The study included 10 low-handicap (LH; 4.3 ± 2.4) and 10 high-handicap (HH; 29.1 ± 5.4) right-handed golfers as participants. The participants were randomly assigned 7-iron clubs with shaft weights categorized as light (77 g), medium (98 g), or heavy (114 g), and they performed test shots. Kinematic data were captured using a motion analysis system with nine infra-red high speed cameras; a force platform connected to this system was used to record weight transfer patterns. Performance variables were assessed using a FlightScope launch monitor. A two-way mixed-design analysis of variance was used to determine the significance of the performance differences among both participant groups and golf shaft weights. The results indicated that during the backswing, the LH group exhibited significantly greater maximum rightward upper torso rotation, maximum X-factor, and maximum right wrist hinge rotation than did the HH group. During the downswing, the LH group exhibited significantly greater maximum upper torso angular velocity and maximum right wrist angular velocity than did the HH group. Moreover, the LH group produced significantly higher ball speeds, longer shot distances, and lower launch angles than did the HH group. The shaft weight neither greatly altered the golf swing nor displaced the center of gravity of the golfers. The lighter shafts were observed to facilitate faster clubhead speeds and initial ball velocities, thereby resulting in longer shot distances, especially among LH golfers. Although significant differences in swing mechanics and performance exist between HH and LH golfers, lighter shafts can contribute to increased shot distances for all golfers.

## 1 Introduction

Golf is an outdoor leisure sport that transcends age, gender, and physical constraints. As of 2021, golf has attracted approximately 66 million participants worldwide (R&A, 2021). Each player aims to enhance shot distance and accuracy to improve performance scores. Haeufle et al. (2012) indicated that improving golf performance involves not only engaging in repetitive practice but also selecting effective golf clubs or clubs tailored to the golfer’s swing specifications. The shaft of a golf club is the component of the club that most substantially affects swing performance, and a shaft’s material properties directly affect its weight and stiffness (Haeufle et al., 2012). Research findings suggest that golfers can optimize their swing and ball flight parameters by suitable selection of drivers with different shaft lengths (Mizoguchi et al., 2005; Kenny et al., 2008; Lacy Jr et al., 2012), flexibility levels (MacKenzie and Sprigings, 2009; Worobets and Stefanyshyn, 2012), kick points (Joyce et al., 2016), and weights (White, 2006; Haeufle et al., 2012; Lacy Jr et al., 2012). However, related research for selection of iron clubs is limited.

Several studies have explored the impact of driver shaft weight on swing performance (White, 2006; Haeufle et al., 2012; Lacy Jr et al., 2012). White (2006) indicated that reducing the shaft weight of a driver by 10% leads to an increase of 1.5 m in driving distance, based on his non-driven double pendulum model. Haeufle et al. (2012) let 12 golfers hit balls with a standard driver and a driver fitted with the same 22 g increase in mass. Ten golfers maintained their clubhead speed, while one experienced a decrease (1.4%), and another demonstrated an increase (3.0%) in clubhead speed due to the additional mass on the club. The authors then concluded that golfers do not respond to changes in club mass in a mechanically predictable way. Lacy Jr et al. (2012) found that lighter commercial drivers generally increased ball speeds for the golfers they tested, but the drivers had varying lengths. This led to the conclusion that combinations of club mass and length, which minimized spin rates, resulted in the greatest estimated total shot distance. Although the effects of shaft weights on golf swing performance were reported, these studies only tested drivers, and the findings were not consistent.

**FIGURE 1:**
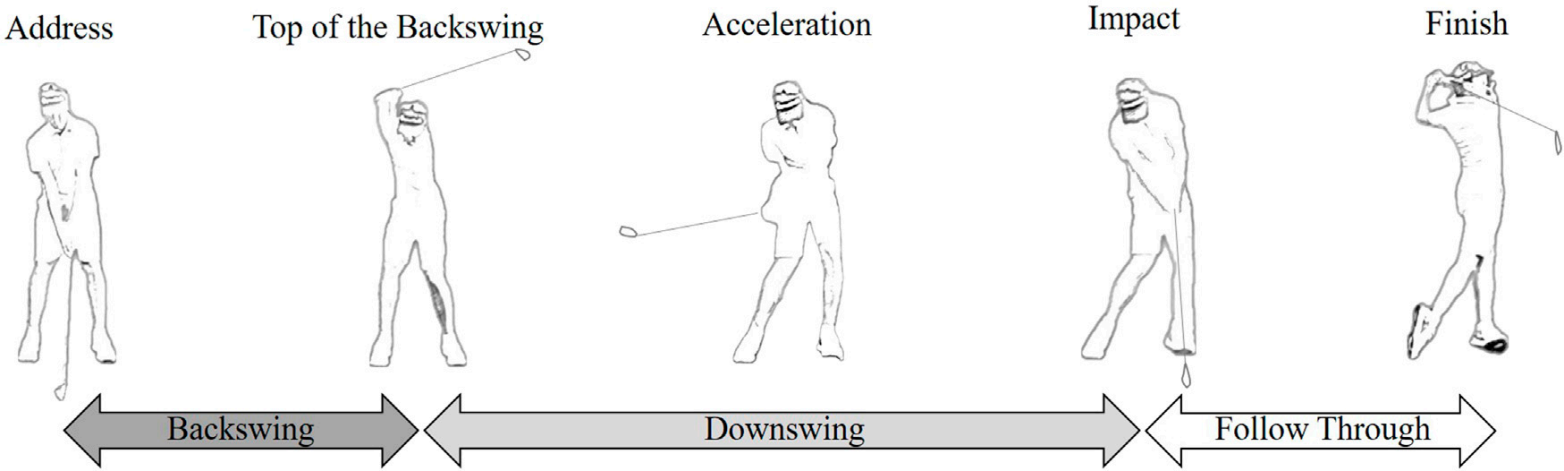
Phases of a golf swing.

**FIGURE 2:**
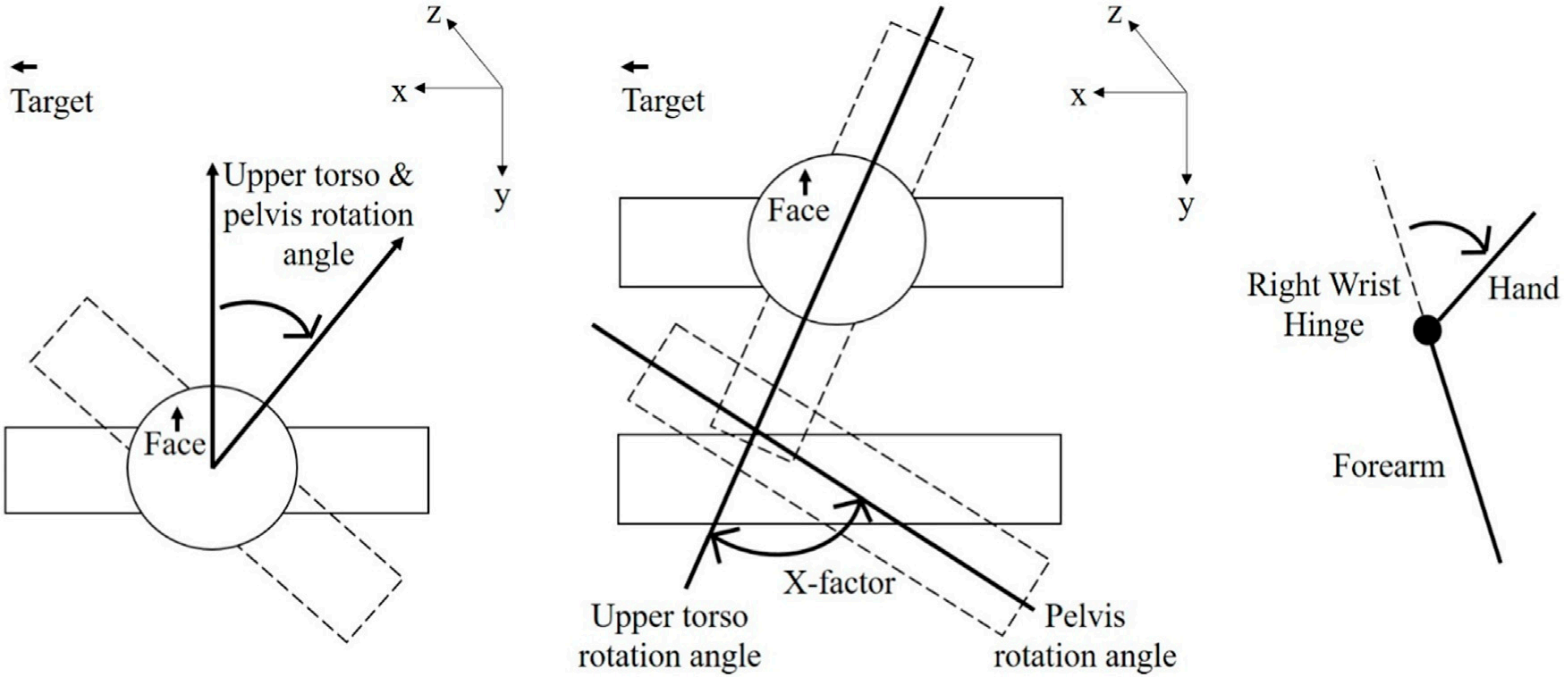
Definitions of rotational angles of the upper torso, pelvis, X-factor, and right wrist.

Studies have explored how golfers’ body movements influence key shot parameters. Biomechanical analyses have provided valuable insights regarding the effect of body movements on clubhead speed during a golf swing. The primary factors influencing clubhead speed are associated with the upper body’s rotation and the lower body’s weight transfer. Zheng et al. (2008) indicated that professional golfers achieved a greater trunk rotation at the top of their backswing (Figure 1) than did amateurs. The trunk rotation, which is the maximum separation angle between the shoulders and pelvis (also known as X-factor) (Figure 2), has been identified as a critical factor influencing clubhead speed (Mclean and Andrisani, 1997; Zheng et al., 2008; Gould et al., 2021; McHugh et al., 2023). A greater X-factor contributes to higher clubhead or ball speeds during impact, thereby resulting in longer shot distances (Cheetham et al., 2001; Myers et al., 2008; Chu et al., 2010; McHugh et al., 2023). Furthermore, during the backswing, as the torso rotates, the rear foot bears an increased weight load, which then shifts to the lead foot in the downswing phase. This weight transfer is instrumental in transferring power to the clubhead to maximize shot distance (Hume et al., 2005). Richards et al. (1985) emphasized the importance of weight transfer patterns and timing within a swing. Golfers with lower handicaps (A handicap represents the skill level of a golfer, with a smaller value indicating superior skill) tend to have more weight on the back heel at impact, whereas those with higher handicaps are more likely to have their weight on the toes. The distribution of weight between the heel and the toes at impact can predict a golfer’s skill level with up to 85% accuracy (Richards et al., 1985). Many studies have confirmed that golfers with lower handicaps tend to transfer their center of gravity (COG) from the rear foot to the lead foot more promptly and generate greater ground reaction forces than do their higher-handicap counterparts during their downswing (Chu et al., 2010; Okuda et al., 2010; Queen et al., 2013). The exploration of the influence of club shaft weight on golf swing mechanics and performance among different skill levels of golfers is relatively uncommon in existing research.

Most of the aforementioned studies have primarily focused on analyzing players using drivers. Experiments by Lindsay et al. (2002) revealed differences in the swing movements between players using a 7-iron club and those using a driver. Specifically, players using the 7-iron club exhibited considerably greater trunk flexion, left-side bend range of motion, and right-side bend velocity. Different types of clubs can also cause different levels of leg stiffness. Studies have reported that the horizontal reaction force created by the lead foot or the trail foot is greater when a driver is used instead of a 6-iron club (Peterson et al., 2016; You et al., 2023). However, iron clubs, which typically have shorter and heavier shafts than do wooden clubs, prioritize precision for landing the ball on the green over maximizing shot distance. In addition, statistical analysis of golfers on the Professional Golfers’ Association (PGA) Tour revealed that the ability to hit the green in regulation, which is typically achieved with an iron club, had a stronger correlation with a player’s earnings than did driving distance with a driver (Wiseman and Chatterjee, 2006). This finding underscores the importance of precision in professional play with iron clubs. Among all iron clubs, golfers often choose the 7-iron to assess how a particular club feels during the swing, impact, and follow-through in golf equipment stores. It allows them to evaluate factors such as the club’s weight, balance, and overall comfort. Furthermore, the 7-iron is a frequently used mid-iron with a moderate length. If a player performs well with this club, it usually indicates that they have good precision and overall proficiency in using other irons as well.

To enhance the practicality of a golf club, the primary consideration regarding club components should be the club weight, ensuring that a player can comfortably handle the weight. Shaft flex or swing weight (weight balance between the clubhead, shaft, and grip) can then be tailored to achieve the desired shot performance and feel (Shan et al., 2008). Whether the use of clubs that are heavier or lighter for one’s skill level can result in the development of specific movement patterns that may affect athletic performance is not clear, particularly when an iron club is used. Accordingly, the present study aimed to explore the effects of three iron shaft weights on swing mechanics, weight transfer, and ball flight parameters among golfers of varying skill levels. The findings could help to broaden the current limited understanding regarding these effects and provide practical insights and recommendations for players, coaches, and club fitters with respect to choosing clubs for golfers of any skill level.

## 2 Materials and methods

### 2.1 Participants

Twenty male right-handed amateur golfers aged between 20 and 30 were invited to participate in this study. Ten of them reported to have a handicap lower than 10 (low-handicap group, LH) and the other ten golfers reported to have a handicap between 16 and 36 (high-handicap group, HH). The handicap levels of these golfers were determined in accordance with the guidelines set forth by the United States Golf Association (USGA) (USGA, 2023). All golfers should not have a history of muscle or joint injuries in the 6 months preceding the study experiment. Table 1 presents details regarding the demographic characteristics of the two groups. All participants were fully briefed on the experimental procedures and associated risks, and they signed an informed consent form approved by the Human Ethics Committee of the University of Taipei.

**TABLE 1:** Demographic characteristics of the participants.

	Low-handicap (LH)		High-handicap (HH)		*p*-Value
	Mean	SD	Mean	SD	
Height (cm)	178.0	6.3	174.5	3.9	0.150
Weight (kg)	77.2	12.0	70.6	8.4	0.170
Age (yr)	21.2	1.0	25.3	3.4	0.002*
Handicap	4.3	2.4	29.1	5.4	<0.001*
Experience (yr)	8.7	1.7	2.7	2.8	<0.001*

Note: * indicates a significant difference (*p <* 0.05).

### 2.2 Instrumentation

The test clubs used in this study were selected from steel shaft brands (N.S. PRO, Nippon Shaft Co., LTD., Numazu, Japan) commonly available on the market. Specifically, three assembled 7-iron clubs with shaft weights of 77 g (light weight), 98 g (medium weight), and 114 g (heavy) separately, which are typically preferred by male golfers, were selected for this study. The clubheads (PING 425, PING Inc., Phoenix, United States), shaft lengths, and grips were standardized across all clubs. Table 2 lists relevant club specifications.

**TABLE 2:** Characteristics of the test clubs.

Club head	Steel shaft	Flex	Grip (g)	Swing weight
PING G425	N.S. PRO Zelos 7 (77 g)	S	50	C8
PING G425	N.S. PRO 950 GH (98 g)	S	50	D0.5
PING G425	N.S. PRO Modus^3^ 120 (114 g)	S	50	D1

Golf swing kinematics were measured using a motion analysis system equipped with nine Raptor-E digital cameras (Motion Analysis Corporation, Santa Rosa, CA, United States). This system was used to capture data at a sampling frequency of 200 Hz. The captured data were processed using Cortex 3.0 motion tracking software (Motion Analysis Corporation, Santa Rosa, CA, United States) to analyze the maximum rotational angles and angular velocities of the upper torso, pelvis, X-factor, and right wrist throughout a swing. The X-factor angle (upper torso-pelvic separation) was calculated by subtracting the pelvic rotation angle from the upper torso rotation angle (Myers et al., 2008). The right wrist hinge angle was calculated as the right wrist extension angle between the forearm and the distal direction of the club shaft (Figure 2). The maximum rotational angles of the upper torso, pelvis, X-factor, and right wrist hinge were captured at the top of the backswing phase, and their maximum angular velocities were captured from downswing to impact (Figure 1). These parameters are generally considered factors that markedly influence golf performance (Myers et al., 2008; Zheng et al., 2008; Chu et al., 2010; McHugh et al., 2023).

To measure COG displacement during a swing, a force plate (AMTI, Watertown, MA, United States) with a data sampling frequency of 1000 Hz was synchronized with the motion analysis system by using Cortex 3.0 motion tracking software (Motion Analysis Corporation, Santa Rosa, CA, United States). Data regarding the following parameters were analyzed: horizontal COG displacement from the address position to the farthest point toward the right foot, displacement from the address position to the farthest point toward the left foot, displacement in the medial-lateral direction, and displacement in the anterior-posterior direction.

Ball flight and clubhead parameters were measured using a FlightScope Xi Launch Monitor (FlightScope Ltd., Orlando, FL, United States) equipped with Doppler radar tracking technology. Images were simulated and then projected onto a hitting screen 2.4 m from the ball’s position, and the launch monitor was positioned 2.4 m behind the ball. Previous studies have established the validity of Doppler radar launch monitor data, showing strong agreement with high-speed video cameras (GOM system) (Leach et al., 2017). Studies have also confirmed the reliability of various parameters influencing golf performance by utilizing the FlightScope system. These parameters include clubhead speed (ICC greater than 0.87) (Read et al., 2013; Ichikawa et al., 2022; Villarrasa-Sapina et al., 2022), ball speed (ICC: 0.89–0.98), carry distance (ICC: 0.86–0.97) (the distance a golf ball travels through the air from the point of impact with the clubface until it first makes contact with the ground), and total distance (ICC: 0.86–0.98) (a combination of carry distance and the estimated rolling distance after landing) for both 6-iron and driver, and launch angle (the angle of the ball relative to the horizontal plane at impact) (ICC: 0.86–0.93) for 6-iron (Villarrasa-Sapina et al., 2022).

### 2.3 Data collection procedures

**FIGURE 3:**
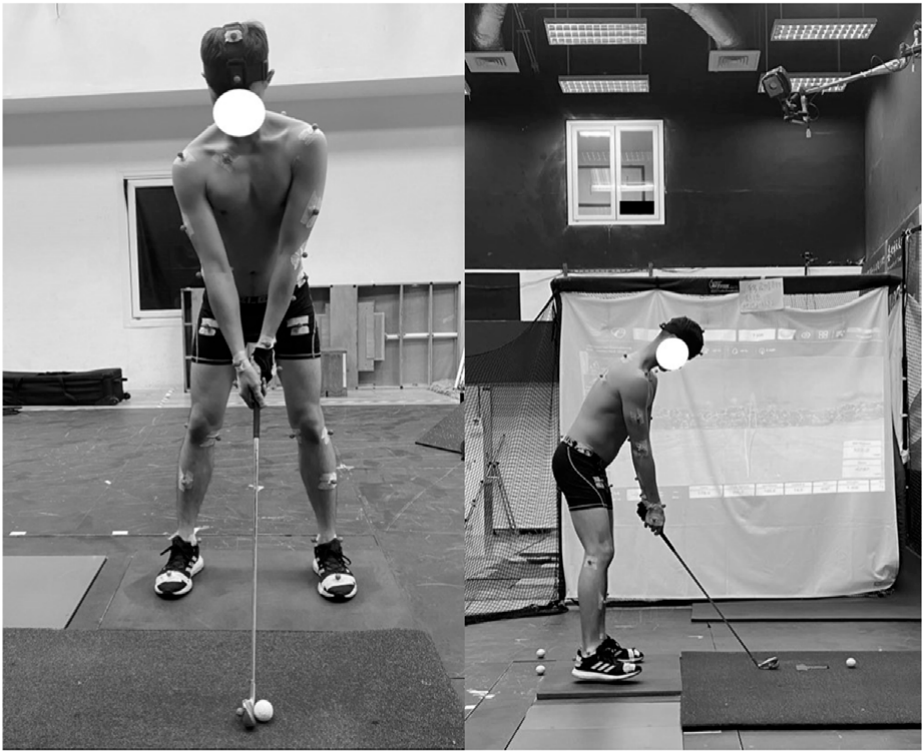
Reflective markers on a golfer in a sports biomechanics laboratory.

This study was conducted in a sports biomechanics laboratory. The participants commenced with a 5-min warm-up to ensure general flexibility. Reflective markers were then placed on their bodies at multiple locations (top head, front head, rear head, right clavicle, sacrum, bilateral sides of the acromion, medial elbow, lateral elbow, medial wrist, lateral wrist, hand, anterior superior iliac spine, posterior superior iliac spine, mid-lateral thigh, medial knee epicondyle, lateral knee epicondyle, mid-lateral shank, medial ankle malleolus, lateral ankle malleolus, and second metatarsal and heel attached to the shoes). One other marker was placed on the club head to record the trajectory of the club (Figure 3). The participants were then randomly assigned one of three 7-iron clubs of varying shaft weights (77, 98, and 114 g). They stood with both feet on the force plate and were instructed to hit balls toward a net (20.3 × 20.8 m^2^) provided by Net Return (Fair Lawn, NJ, United States), which featured a practice range image screen. Fifteen practice shots were allowed with each iron to acclimatize the participants to the equipment. After a 5-min interval, they executed five test shots Golf balls (Titleist Pro V1, Acushnet, MA, United States of America) were consistently positioned on an artificial mat (1.5 × 1.5 m^2^). Data on the swing kinematics, weight transfer patterns, and shot performance were collected concurrently with each swing. For each participant, the best two out of five shots were selected for statistical analysis (Zheng et al., 2008).

### 2.4 Statistical analysis

The raw data collected in this study were analyzed using SPSS for Windows (version 25.0; an IBM Corp., United States). The demographic characteristics of the two groups of golfers were compared using independent *t* tests. A two-factor mixed-design analysis of variance (ANOVA) (2 groups × 3 clubs) was conducted to determine differences in swing kinematics, weight transfer patterns, and shot performance between the LH and HH groups when they used the three 7-iron clubs with different shaft weights. The threshold for statistical significance was set at α = 0.05. When a significant interaction effect was observed, *post hoc* tests were conducted to examine simple main effects. Independent *t* tests were conducted to compare the two groups when they used each of the golf clubs, and the Bonferroni method was employed to determine differences in parameters among the three 7-iron club shaft weights for each group of golfers.

## 3 Results

### 3.1 Shot performance

The lightweight shaft resulted in a significantly higher clubhead speed than did the heavy shaft (*p* = 0.022). The ball speeds generated using the lightweight and medium-weight shafts were significantly higher than that generated using the heavy shaft (*p* = 0.004). Furthermore, between-group comparison results demonstrated that the LH group achieved significantly higher ball speeds across the various shaft weights than did the HH group (*p* = 0.001) (Table 3).

**TABLE 3:** Shot performance results of the LH and HH groups across various shaft weights.

	Low-handicap (LH)		High-handicap (HH)		*p‐Value*		
	Mean	SD	Mean	SD	Shafts	Groups	Interaction
Clubhead speed (mph)							
Light-weight	88.74	4.95	83.41	6.12	0.022*	0.052	0.398
Mid-weight	87.84	4.62	83.22	6.74	light > heavy		
Heavy-weight	87.77	4.35	82.09	6.85			
Ball speed (mph)							
Light-weight	128.94	6.34	111.31	11.91	0.004*	0.001*	0.060
Mid-weight	126.70	5.75	111.62	12.27	light > heavy	LH > HH	
Heavy-weight	126.50	5.15	109.15	12.32	mid > heavy		
Carry (yds)							
Light-weight	194.09	14.07	156.17	24.95	0.008*	0.001*	0.062
Mid-weight	189.40	12.55	157.66	25.24	light > heavy	LH > HH	
Heavy-weight	188.38	11.30	151.86	25.32	mid > heavy		
Total distance (yds)							
Light-weight	198.59	14.51	158.78	25.81	0.004*	0.001*	0.029*
Mid-weight	193.49	13.02	160.40	25.77	light > heavy	LH > HH	LH: light > mid, *p* = 0.019
Heavy-weight	192.91	11.57	154.11	26.09	mid > heavy		LH: light > heavy, *p* = 0.014**
Launch angle (°)							
Light-weight	14.70	0.96	18.44	2.47	0.363	<0.001*	0.371
Mid-weight	15.13	0.79	18.71	2.45		HH > LH	
Heavy-weight	14.37	1.05	18.73	2.64			

Note: For the two-way mixed-design ANOVA, * indicates a significant difference (*p <* 0.05); for the Bonferroni correction method used in the *post hoc* comparisons, ** indicates a significant difference (*p <* 0.017).

The lightweight and medium-weight shafts resulted in significantly greater carry distances than did the heavy shaft (*p* = 0.008). Between-group comparison results revealed that the LH group achieved significantly greater carry distances across the various shaft weights than did the HH group (*p* = 0.001). Regarding the total distance, a significant interaction was observed between the various shaft weights and groups (*p* = 0.029). The results of the *post hoc* tests indicated that the lightweight shaft led to a significantly greater total distance than did the heavy shaft (*p* = 0.014) in the LH group. The HH group demonstrated significantly higher launch angles (*p* < 0.001) across the various shaft weights than did the LH group (Table 3).

### 3.2 Kinematic data

The LH group exhibited significantly greater maximum rightward upper torso rotation (*p* = 0.008) and maximum X-factor (*p* = 0.007) during the backswing phase across the iron shaft weights than did the HH group (Table 4). The LH group also exhibited a greater maximum right wrist hinge angle (*p* = 0.004), maximum upper torso angular velocity (*p* = 0.017), and maximum right wrist angular velocity (*p* = 0.026) during the downswing phase than did the HH group. A significant interaction effect among the various shaft weights and groups on the maximum pelvic rotation angular velocity was observed (*p* = 0.024). However, the results of *post hoc* tests revealed no significant differences among the various iron shaft weights in either group or between the groups for any of the iron shaft weights after Bonferroni correction (Table 4).

**TABLE 4:** Results for the swing kinematics of the LH and HH groups across various shaft weights.

	Low-handicap (LH)		High-handicap (HH)		*p-Value*		
	Mean	SD	Mean	SD	Shafts	Groups	Interaction
**Backswing**							
Maximum rightward upper torso rotation (°)							
Light-weight	128.20	19.37	108.19	9.90	0.703	0.008*	0.367
Mid-weight	128.35	19.02	109.02	9.53		LH > HH	
Heavy-weight	128.81	19.19	107.62	10.24			
Maximum rightward pelvis rotation (°)							
Light-weight	49.31	9.77	56.36	10.43	0.680	0.208	0.866
Mid-weight	48.81	8.98	54.26	7.77			
Heavy-weight	48.49	8.59	55.81	10.92			
Maximum X-factor (°)							
Light-weight	83.59	24.74	57.07	13.52	0.553	0.007*	0.762
Mid-weight	84.39	24.29	57.21	13.86		LH > HH	
Heavy-weight	83.79	22.63	56.51	15.51			
Maximum right wrist hinge (°)							
Light-weight	72.75	8.21	58.07	9.60	0.714	0.004*	0.341
Mid-weight	70.90	7.86	59.59	10.39		LH > HH	
Heavy-weight	72.17	7.93	60.17	11.06			
**Downswing**							
Maximum upper torso angular velocity (°/s)							
Light-weight	710.30	25.17	659.57	79.91	0.685	0.017*	0.235
Mid-weight	697.78	45.40	658.38	62.62		LH > HH	
Heavy-weight	710.37	56.03	681.61	54.50			
Maximum pelvis angular velocity (°/s)							
Light-weight	484.33	45.98	431.95	75.96	0.286	0.310	0.024*
Mid-weight	470.16	46.92	465.82	108.47			LH: light > mid, *p* = 0.031
Heavy-weight	491.42	59.83	451.85	76.62			LH: heavy > mid, *p* = 0.047
Maximum X-factor angular velocity (°/s)							
Light-weight	576.34	208.22	459.20	75.40	0.690	0.190	0.235
Mid-weight	578.45	207.34	484.24	110.87			
Heavy-weight	544.80	167.94	490.35	86.74			
Maximum right wrist angular velocity (°/s)							
Light-weight	745.35	166.12	580.18	156.11	0.854	0.026*	0.832
Mid-weight	764.42	213.96	585.74	143.30		LH > HH	
Heavy-weight	778.15	224.28	578.10	180.25			

Note: For the two-way mixed-design ANOVA, * indicates a significant difference (*p <* 0.05); for the Bonferroni correction method used in the *post hoc* comparisons, ** indicates a significant difference (*p <* 0.017).

### 3.3 COG data

The HH group demonstrated significantly greater rightward COG displacements across the various shaft weights than did the LH group (*p* = 0.002; Table 5). No significant differences were observed in leftward COG displacement, medial-lateral displacement, or anterior-posterior displacement between the two handicap groups across the various shaft weights.

**TABLE 5:** Center of gravity (COG) displacements of the LH and HH groups across various shaft weights.

	Low-handicap (LH)		High-handicap (HH)		*p-Value*		
	Mean	SD	Mean	SD	Shafts	Groups	Interaction
COG from address position to the left (mm)							
Light-weight	122.30	15.55	111.44	35.94	0.162	0.156	0.078
Mid-weight	124.81	13.93	103.34	30.94			
Heavy-weight	121.93	18.65	101.53	36.27			
COG from address position to the right (mm)							
Light-weight	30.55	11.02	63.83	20.91	0.905	0.002*	0.296
Mid-weight	32.43	13.11	57.66	17.69		HH > LH	
Heavy-weight	33.69	11.73	61.26	21.80			
Medial-lateral displacement range (mm)							
Light-weight	157.70	18.87	164.17	44.02	0.375	0.860	0.047
Mid-weight	161.80	15.99	156.05	36.77			
Heavy-weight	160.67	20.71	152.64	40.21			
Anterior-posterior displacement range (mm)							
Light-weight	37.66	8.22	51.47	27.29	0.602	0.315	0.373
Mid-weight	38.18	12.31	44.90	20.99			
Heavy-weight	36.98	10.47	45.41	15.67			

Note: for the two-way mixed-design ANOVA, * indicates a significant difference (*p* < 0.05).

## 4 Discussion

The aim of this study was to investigate differences in swing kinematics, weight transfer, and shot performance among amateur golfers of varying skill levels when they used iron clubs with different shaft weights. The results revealed that a lighter shaft considerably enhanced clubhead speed and ball speed, leading to increased shot distance. This effect was particularly pronounced in the LH group. The weight of the iron shaft did not significantly alter the swing mechanics and the COG shift pattern for the golfers.

In this study, we tested 7-iron clubs with shaft weights of 77, 98, and 114 g in an indoor environment. The effects of different shaft weights on clubhead speed, ball speed, and carry distance were similar for both groups of golfers. These golfers demonstrated faster clubhead speed, ball speed, and longer carry distance with a lighter shaft. The findings differ from those reported by Haeufle et al. (2012). In their study, most golfers (with handicaps between −1 and 16) did not exhibit significant changes in clubhead speed when using a driver with an additional 22 g on the shaft. While Lacy Jr et al. (2012) reported that lighter commercial drivers generally resulted in higher ball speeds for golfers, the drivers they used varied in length. Consequently, they could not conclusively attribute the higher ball speed solely to the lighter shaft weight. From the above mentioned research findings, it appears that the distinct characteristics of iron club and driver may contribute to varied effects of shaft weight on the shot performance of golfers. Based on the mentioned research findings, it appears that the distinct characteristics of iron clubs and drivers may contribute to varied effects of shaft weight on golfers’ shot performance. Irons offer more control and precision, making them suitable for hitting mid-short distances on the course, while drivers are designed to provide greater flight distance, mainly for tee shots.

We observed that the mean clubhead speed and ball speed of the LH group were significantly higher than that of the HH group. The results were similar to those of Myers et al. (2008) and Healy et al. (2011), who used a driver and 5-iron club, respectively, and observed that groups with higher ball speeds had lower handicaps. This may be because LH golfers usually have better swing mechanics that can generate faster clubhead speed and ball speed, leading to a longer driving distance. In addition, the LH group exhibited significantly lower launch angles compared to the HH group in this study. Wallace et al. (2007) also indicated that golfers with single-digit-handicap demonstrated a significant negative relationship between launch angle and ball speed although they used drivers with different lengths in their study (Wallace et al., 2007). The findings of both studies suggest that higher ball speeds may be associated with lower launch angles for increasing driving distance in elite golfers no matter whether drivers or iron clubs are used. As for the effects of shaft weight, the LH group achieved significantly greater total distances when they used the lightweight shaft than when they used the heavy shaft in this study. A trend toward increased total distance was also noted with the lightweight shaft as opposed to the medium-weight shaft. For the HH group, the effect of shaft weight on total distance was less significant, which suggested that the benefits of different shaft weights were less pronounced because of variations in swing techniques and skill levels.

Several studies have indicated that LH golfers demonstrate superior swing performance compared to HH golfers (Cheetham et al., 2001; Myers et al., 2008; Zheng et al., 2008). This result is primarily attributed to trunk, pelvic, and wrist movements, as well as to X-factor, during a swing. Myers et al. (2008) indicated that although maximum rotation in the upper torso and pelvis did not significantly affect ball speed, maximum torso-pelvic separation (X-factor), maximum X-factor angular velocity, maximum upper torso rotation velocity, and maximum pelvic rotation velocity were highly correlated with ball speed and differed significantly between the high-ball-speed (handicap: 1.8 ± 3.2) and low-ball-speed (handicap: 15.1 ± 5.2) groups. The present study showed that the LH group exhibited a significantly greater maximum rightward trunk rotation angle, maximum X-factor, and maximum trunk rotation angular velocity than did the HH group across the various shaft weights. The difference in maximum rightward trunk rotation angle between the aforementioned studies can be attributed to the differences in participant handicap ranges between these studies. For example, in Myers et al. (2008), the HH group, which had lower ball speed compared with that of the LH group, had a handicap of 15.1 ± 5.2; by contrast, the HH group in the present study had a handicap of 29.1 ± 5.4. Zheng et al. (2008) also demonstrated that professional and amateur golfers with a handicap lower than 10 exhibited significantly greater maximum trunk rotation angles than did their HH counterparts (21.3 ± 3.8). Golfers with higher handicaps often exhibit insufficient trunk rotation, potentially limiting their ability to fully optimize shot effectiveness. The wrist joint is another critical factor influencing clubhead speed (Robinson, 1994). A larger wrist hinge angle at the top of the backswing and the quickest wrist release during the final acceleration phase, particularly in the last 40 m before impact, contribute to an increased clubhead speed (Sprigings and Neal, 2000; Chu et al., 2010). Similarly, Zheng et al. (2008) demonstrated that professional and amateur golfers with a handicap lower than 10 exhibited significantly higher maximum angular velocities of the right wrist during the downswing phase than did HH golfers. The present study revealed that the LH group demonstrated a greater right wrist hinge angle during the backswing and significantly higher maximum angular velocities of the wrist during the downswing, which are consistent with previous research findings.

In the present study, the use of clubs with three different 7-iron shaft weights did not yield any significant differences in the maximum wrist hinge, upper torso, pelvis, or X-factor angles during the backswing, as well as in the maximum angular velocity of those angles during the downswing. This finding demonstrates that golfers enhanced their shot distance by using lighter iron clubs without altering their major swing patterns. Unlike the kinematic parameters assessed in our study, Joyce et al. (2016) reported that a driver with a 56-g shaft facilitated an earlier wrist release during the downswing phase and thus accelerated trunk axial rotation upon impact and produced a higher launch angle (by 2°) compared with that of a driver with a 78-g shaft. The differences between the findings of the present study and those of Joyce et al. (2016) could be attributed to the disparity in shaft length between the driver and the 7-iron club, which could affect swing mechanics. Notably, the findings of Joyce et al. (2016) might have resulted from carbon fiber shafts being more flexible and lighter than steel iron shafts and thus affecting the golf swing.

Regarding COG displacement during a swing, this study observed that the HH group exhibited a more significant rightward COG displacement than did the LH group across the various shaft weights. Okuda et al. (2010) indicated that LH golfers exhibited significantly increased trunk and pelvic rotation and higher vertical ground reaction forces generated by the rear foot during the backswing phase compared to HH players. At the top of the backswing, the LH players had already reduced their weight transfer by 18% of the body weight, whereas the HH players increased their weight transfer by 7% of the body weight. Consistent with these findings, the present study noted that the HH group exhibited a greater rightward COG displacement during the backswing. This trend is consistent with common errors observed among HH golfers. However, previous research has not extensively investigated the effects of club composition on COG displacement during golf swings across different handicap levels. In our study, the three 7-iron shaft weights did not result in notable changes in medial-lateral or anterior-posterior COG displacement in the participants. This finding aligns with our observations in kinematic parameters. Since there were no significant alterations in the swing motion among the three 7-iron shaft weights, corresponding changes in weight shift were also not significant.

This study has several limitations that warrant consideration. First, owing to the limited sample size, the results are not generalizable to the general population. The LH group in this study was younger but had more years of experience than did the HH group. Experience is a key factor if golfers intend to become single-digit-handicap golfers. In addition to developing an effective swing, golfers must acquire skills related to strategy and club usage. Studies that have investigated golf handicaps have observed that a golfer’s handicap has a strong correlation with their level of experience (Fedorcik et al., 2012; Okuda et al., 2010). Although this correlation is not universal, most skilled golfers are experienced and able to better adapt to changes in golf clubs. Second, to enhance the practicality of the golf clubs for our participants, we selected common iron shaft brands and weights favored by male golfers in the marketplace. Although the clubhead, grip, and shaft length were standardized, other variables, such as shaft flex and swing weight, were not independently assessed. Nevertheless, a previous study demonstrated that varying stiffness levels of a 5-iron shaft do not significantly affect clubhead speed, swing path angle, and shoulder rotation angle for golfers (Wallace and Hubbell, 2001). Furthermore, this study did not allow the participants to use their own golf clubs. However, the weight of each golfer’s 7-iron club fell within the spectrum of weights tested in this study.

## 5 Conclusion

The findings of this study indicate that the primary effect of iron shaft weight on swing performance is related to shot distance. The use of lighter shafts results in higher clubhead speed and initial ball speed, which can lead to longer shot distances. Variations in shaft weight do not significantly affect swing mechanics or COG displacement. Golfers with lower handicaps possess superior swing adjustment capabilities (Herder and Benoit, 2022), resulting in more pronounced benefits in shot performance. Although the swing mechanics of HH golfers are significantly different from those of LH golfers, the use of lighter shafts should still contribute to increased shot distances.

## Data Availability

The raw data supporting the conclusion of this article will be made available by the authors, without undue reservation.
